# Methyl 2-(4-meth­oxy-3-nitro­benzamido)­acetate

**DOI:** 10.1107/S1600536812029522

**Published:** 2012-07-04

**Authors:** Xin Zhu, Qiujuan Ma

**Affiliations:** a College of Pharmacy, Henan University of Traditional Chinese Medicine, Zhengzhou, Henan 450008, People’s Republic of China

## Abstract

The title compound, C_11_H_12_N_2_O_6_, crystallizes with two independent mol­ecules in the asymmetric unit, which differ slightly in conformation. The dihedral angle between the amide O=C—N plane and the attached benzene ring is 19.5 (3)° in one mol­ecule and 23.4 (3)° in the other. In the crystal, the two independent mol­ecules are connected alternately by N—H⋯O hydrogen bonds, forming a chain along the *a* axis.

## Related literature

For the biological activity of compounds with nitro and ester groups, see: Sykes *et al.* (1999 ▶). For a related structure, see: Wu *et al.* (2011 ▶).
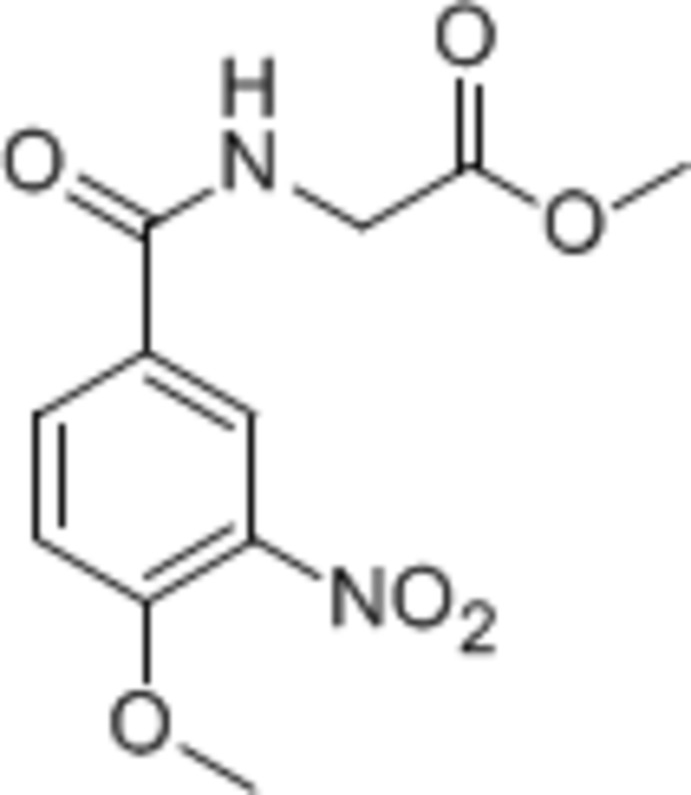


## Experimental

### 

#### Crystal data

C_11_H_12_N_2_O_6_
*M* *_r_* = 268.23
Monoclinic, 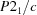

*a* = 10.4378 (7) Å
*b* = 13.9110 (9) Å
*c* = 17.5420 (15) Å
β = 106.146 (8)°
*V* = 2446.6 (3) Å^3^
*Z* = 8
Cu *K*α radiation
μ = 1.04 mm^−1^
*T* = 291 K
0.28 × 0.26 × 0.24 mm

#### Data collection

Agilent Xcalibur Eos Gemini diffractometer
Absorption correction: multi-scan (*CrysAlis PRO*; Agilent, 2011 ▶) *T*_min_ = 0.760, *T*_max_ = 0.789
10600 measured reflections
4251 independent reflections
3287 reflections with *I* > 2σ(*I*)
*R*_int_ = 0.026

#### Refinement

*R*[*F* ^2^ > 2σ(*F* ^2^)] = 0.052
*wR*(*F* ^2^) = 0.155
*S* = 1.04
4251 reflections
356 parameters
2 restraints
H atoms treated by a mixture of independent and constrained refinement
Δρ_max_ = 0.50 e Å^−3^
Δρ_min_ = −0.31 e Å^−3^

### 

Data collection: *CrysAlis PRO* (Agilent, 2011 ▶); cell refinement: *CrysAlis PRO*; data reduction: *CrysAlis RED* (Agilent, 2011 ▶); program(s) used to solve structure: *SHELXS97* (Sheldrick, 2008 ▶); program(s) used to refine structure: *SHELXL97* (Sheldrick, 2008 ▶); molecular graphics: *OLEX2* (Dolomanov *et al.*, 2009 ▶); software used to prepare material for publication: *OLEX2*.

## Supplementary Material

Crystal structure: contains datablock(s) global, I. DOI: 10.1107/S1600536812029522/is5151sup1.cif

Structure factors: contains datablock(s) I. DOI: 10.1107/S1600536812029522/is5151Isup2.hkl

Supplementary material file. DOI: 10.1107/S1600536812029522/is5151Isup3.cml

Additional supplementary materials:  crystallographic information; 3D view; checkCIF report

## Figures and Tables

**Table 1:** Hydrogen-bond geometry (Å, °)

*D*—H⋯*A*	*D*—H	H⋯*A*	*D*⋯*A*	*D*—H⋯*A*
N2—H2⋯O4′^i^	0.83 (2)	2.26 (2)	3.069 (2)	167 (2)
N2′—H2′⋯O4	0.83 (2)	2.23 (2)	3.016 (2)	158 (2)

Symmetry code: (i) 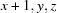.

**Fig. 1.**
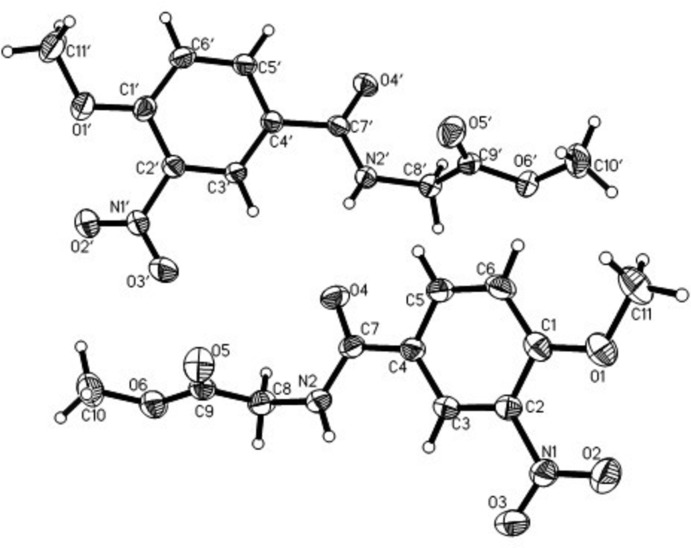
A view of the molecular structure of the title compound The displacement ellipsoids are drawn at the 30% probability level.

**Fig. 2.**
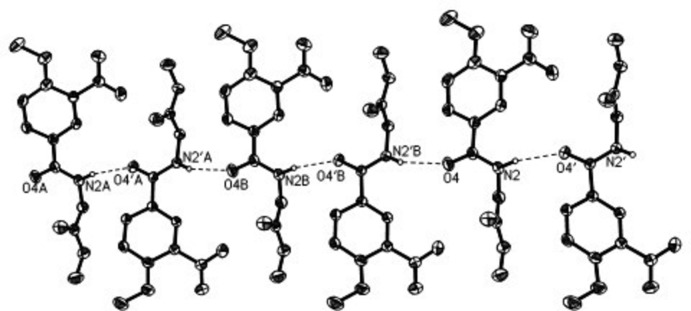
A view of the hydrogen bonded polymeric chain. The hydrogen bonds are shown as dashed lines.

## supplementary crystallographic information

### Comment

The nitro group and ester group are present in many bioactive compounds or be used as prodrug (Sykes *et al.*, 1999). Here we report the crystal structure of C_11_H_12_N_2_O_6_, namely, methyl 2-(4-methoxy-3-nitrobenzamido)acetate. In the crystal of the title compound (Fig. 1), the carbonyl group and the adjacent amide group are connected by intermolecular N—H···O hydrogen bonds, forming a chain along the *a* axis (Fig. 2).

### Experimental

The title compound (0.25 mmol, 67.0 mg) was dissolved in water-methanol (1 ml / 2 ml *v*/*v*) mixture. Colorless block crystals were separated after several weeks.

### Refinement

N-bound H atoms were located in a difference Fourier map and refined freely with a bond-distance restraint of N—H = 0.86 (2) Å. C-bound H-atoms were included in calculated positions and treated as riding atoms, with C—H = 0.93, 0.96 and 0.98 Å for CH(aromatic), CH_3_ and CH(methine) H-atoms, respectively, and with *U*_iso_(H)= k×*U*_eq_(parent C-atom), where k = 1.5 for CH_3_ H-atoms and k = 1.2 for other H-atoms.

### Crystal data

**Table:**

C_11_H_12_N_2_O_6_	*F*(000) = 1120
*M**_r_* = 268.23	*D*_x_ = 1.456 Mg m^−^^3^
Monoclinic, *P*2_1_/*c*	Cu *K*α radiation, λ = 1.54184 Å
Hall symbol: -P 2ybc	Cell parameters from 3206 reflections
*a* = 10.4378 (7) Å	θ = 3.2–67.0°
*b* = 13.9110 (9) Å	µ = 1.04 mm^−^^1^
*c* = 17.5420 (15) Å	*T* = 291 K
β = 106.146 (8)°	Block, colorless
*V* = 2446.6 (3) Å^3^	0.28 × 0.26 × 0.24 mm
*Z* = 8	

### Data collection

**Table:**

Agilent Xcalibur Eos Gemini diffractometer	4251 independent reflections
Radiation source: fine-focus sealed tube	3287 reflections with *I* > 2σ(*I*)
Graphite monochromator	*R*_int_ = 0.026
ω scans	θ_max_ = 67.0°, θ_min_ = 4.1°
Absorption correction: multi-scan (*CrysAlis PRO*; Agilent, 2011)	*h* = −12→12
*T*_min_ = 0.760, *T*_max_ = 0.789	*k* = −16→16
10600 measured reflections	*l* = −20→15

### Refinement

**Table:**

Refinement on *F*^2^	Secondary atom site location: difference Fourier map
Least-squares matrix: full	Hydrogen site location: inferred from neighbouring sites
*R*[*F*^2^ > 2σ(*F*^2^)] = 0.052	H atoms treated by a mixture of independent and constrained refinement
*wR*(*F*^2^) = 0.155	*w* = 1/[σ^2^(*F*_o_^2^) + (0.073*P*)^2^ + 0.8379*P*] where *P* = (*F*_o_^2^ + 2*F*_c_^2^)/3
*S* = 1.04	(Δ/σ)_max_ = 0.004
4251 reflections	Δρ_max_ = 0.50 e Å^−^^3^
356 parameters	Δρ_min_ = −0.31 e Å^−^^3^
2 restraints	Extinction correction: *SHELXL97* (Sheldrick, 2008), Fc^*^=kFc[1+0.001xFc^2^λ^3^/sin(2θ)]^-1/4^
Primary atom site location: structure-invariant direct methods	Extinction coefficient: 0.00093 (17)

### Special details

**Table:**

Geometry. All e.s.d.'s (except the e.s.d. in the dihedral angle between two l.s. planes) are estimated using the full covariance matrix. The cell e.s.d.'s are taken into account individually in the estimation of e.s.d.'s in distances, angles and torsion angles; correlations between e.s.d.'s in cell parameters are only used when they are defined by crystal symmetry. An approximate (isotropic) treatment of cell e.s.d.'s is used for estimating e.s.d.'s involving l.s. planes.
Refinement. Refinement of *F*^2^ against ALL reflections. The weighted *R*-factor *wR* and goodness of fit *S* are based on *F*^2^, conventional *R*-factors *R* are based on *F*, with *F* set to zero for negative *F*^2^. The threshold expression of *F*^2^ > σ(*F*^2^) is used only for calculating *R*-factors(gt) *etc*. and is not relevant to the choice of reflections for refinement. *R*-factors based on *F*^2^ are statistically about twice as large as those based on *F*, and *R*- factors based on ALL data will be even larger.

### Fractional atomic coordinates and isotropic or equivalent isotropic displacement parameters (Å^2^)

**Table:**

	*x*	*y*	*z*	*U*_iso_*/*U*_eq_	
O1	0.7104 (2)	0.31701 (14)	0.29328 (12)	0.0732 (6)	
O1'	0.18346 (19)	1.11580 (13)	0.30677 (12)	0.0671 (5)	
O2	0.9503 (2)	0.31213 (17)	0.3839 (2)	0.1162 (11)	
O2'	0.4242 (2)	1.12599 (15)	0.40786 (18)	0.0981 (9)	
O3	1.0472 (2)	0.44345 (17)	0.41534 (18)	0.1013 (9)	
O3'	0.5286 (2)	0.99534 (16)	0.41347 (19)	0.1039 (9)	
O4	0.58759 (15)	0.72298 (13)	0.42220 (11)	0.0593 (5)	
O4'	0.09322 (15)	0.69905 (12)	0.43303 (11)	0.0576 (5)	
O5	0.7439 (2)	0.90062 (15)	0.34121 (12)	0.0724 (6)	
O5'	0.2464 (2)	0.54205 (14)	0.32784 (11)	0.0677 (5)	
O6	0.82081 (18)	0.99909 (13)	0.44488 (13)	0.0688 (5)	
O6'	0.33449 (16)	0.43396 (11)	0.42284 (11)	0.0563 (4)	
N1	0.9474 (2)	0.39794 (16)	0.38787 (13)	0.0579 (5)	
N1'	0.4254 (2)	1.04059 (14)	0.39950 (14)	0.0569 (5)	
N2	0.80857 (19)	0.74112 (15)	0.44094 (13)	0.0523 (5)	
N2'	0.31167 (18)	0.68992 (14)	0.43984 (12)	0.0473 (5)	
C1	0.7024 (2)	0.40601 (18)	0.32103 (14)	0.0530 (6)	
C1'	0.1806 (2)	1.02642 (16)	0.33482 (14)	0.0483 (5)	
C2	0.8203 (2)	0.44932 (17)	0.36718 (13)	0.0457 (5)	
C2'	0.3009 (2)	0.98568 (16)	0.38040 (14)	0.0454 (5)	
C3	0.8207 (2)	0.54128 (17)	0.39638 (13)	0.0443 (5)	
H3	0.9007	0.5688	0.4253	0.053*	
C3'	0.3080 (2)	0.89318 (15)	0.40887 (13)	0.0432 (5)	
H3'	0.3896	0.8679	0.4377	0.052*	
C4	0.7030 (2)	0.59338 (17)	0.38321 (13)	0.0458 (5)	
C4'	0.1937 (2)	0.83750 (16)	0.39470 (13)	0.0433 (5)	
C5	0.5861 (2)	0.55008 (19)	0.33762 (15)	0.0545 (6)	
H5	0.5061	0.5836	0.3283	0.065*	
C5'	0.0740 (2)	0.87772 (17)	0.35111 (14)	0.0486 (5)	
H5'	−0.0038	0.8417	0.3423	0.058*	
C6	0.5852 (2)	0.45980 (19)	0.30610 (16)	0.0588 (7)	
H6	0.5058	0.4342	0.2745	0.071*	
C6'	0.0668 (2)	0.96912 (17)	0.32055 (15)	0.0530 (6)	
H6'	−0.0146	0.9930	0.2901	0.064*	
C7	0.6947 (2)	0.69072 (17)	0.41681 (13)	0.0464 (5)	
C7'	0.1950 (2)	0.73673 (16)	0.42440 (13)	0.0426 (5)	
C8	0.8122 (3)	0.83675 (19)	0.47275 (15)	0.0569 (6)	
H8A	0.8988	0.8474	0.5103	0.068*	
H8B	0.7455	0.8418	0.5015	0.068*	
C8'	0.3254 (2)	0.59067 (16)	0.46412 (14)	0.0495 (6)	
H8'A	0.2655	0.5777	0.4964	0.059*	
H8'B	0.4158	0.5799	0.4969	0.059*	
C9	0.7875 (2)	0.91368 (18)	0.41080 (17)	0.0528 (6)	
C9'	0.2959 (2)	0.52156 (16)	0.39587 (14)	0.0458 (5)	
C10	0.7937 (3)	1.0825 (2)	0.3943 (2)	0.0863 (10)	
H10D	0.8540	1.1333	0.4180	0.130*	
H10E	0.7036	1.1033	0.3878	0.130*	
H10F	0.8055	1.0667	0.3434	0.130*	
C10'	0.3005 (3)	0.3553 (2)	0.3677 (2)	0.0757 (9)	
H10A	0.3653	0.3050	0.3839	0.114*	
H10B	0.2997	0.3773	0.3156	0.114*	
H10C	0.2138	0.3312	0.3666	0.114*	
C11	0.5910 (4)	0.2707 (2)	0.2494 (2)	0.0936 (11)	
H11D	0.5337	0.2608	0.2831	0.140*	
H11E	0.6125	0.2098	0.2304	0.140*	
H11F	0.5463	0.3103	0.2052	0.140*	
C11'	0.0631 (3)	1.1560 (2)	0.25777 (19)	0.0801 (9)	
H11A	−0.0002	1.1630	0.2880	0.120*	
H11B	0.0270	1.1143	0.2134	0.120*	
H11C	0.0814	1.2179	0.2389	0.120*	
H2'	0.3781 (19)	0.7135 (17)	0.4293 (14)	0.049 (7)*	
H2	0.8799 (19)	0.7210 (17)	0.4351 (15)	0.049 (7)*	

### Atomic displacement parameters (Å^2^)

**Table:**

	*U*^11^	*U*^22^	*U*^33^	*U*^12^	*U*^13^	*U*^23^
O1	0.0720 (12)	0.0635 (12)	0.0763 (13)	−0.0131 (10)	0.0076 (10)	−0.0211 (10)
O1'	0.0693 (11)	0.0466 (10)	0.0737 (12)	0.0043 (9)	0.0004 (9)	0.0127 (9)
O2	0.0815 (16)	0.0625 (14)	0.180 (3)	0.0145 (12)	−0.0038 (17)	−0.0290 (16)
O2'	0.0691 (13)	0.0446 (12)	0.163 (2)	−0.0081 (10)	0.0032 (14)	0.0035 (13)
O3	0.0439 (11)	0.0780 (15)	0.173 (3)	0.0028 (10)	0.0148 (13)	−0.0273 (16)
O3'	0.0447 (11)	0.0661 (14)	0.199 (3)	−0.0027 (10)	0.0301 (14)	0.0091 (15)
O4	0.0399 (8)	0.0660 (11)	0.0736 (11)	0.0104 (8)	0.0183 (8)	0.0060 (9)
O4'	0.0395 (8)	0.0518 (10)	0.0837 (12)	−0.0066 (7)	0.0210 (8)	0.0000 (9)
O5	0.0894 (14)	0.0647 (12)	0.0632 (12)	−0.0003 (11)	0.0215 (11)	−0.0005 (9)
O5'	0.0811 (13)	0.0605 (11)	0.0553 (11)	0.0090 (10)	0.0086 (10)	0.0041 (9)
O6	0.0583 (10)	0.0522 (11)	0.0941 (14)	−0.0040 (8)	0.0180 (10)	−0.0118 (10)
O6'	0.0528 (9)	0.0393 (9)	0.0741 (11)	0.0058 (7)	0.0132 (8)	0.0056 (8)
N1	0.0502 (12)	0.0539 (13)	0.0684 (13)	0.0031 (10)	0.0143 (10)	−0.0125 (10)
N1'	0.0493 (11)	0.0408 (11)	0.0788 (15)	−0.0036 (9)	0.0149 (10)	0.0020 (10)
N2	0.0395 (10)	0.0490 (12)	0.0679 (13)	0.0062 (9)	0.0140 (9)	−0.0024 (10)
N2'	0.0375 (9)	0.0394 (10)	0.0666 (13)	−0.0009 (8)	0.0168 (9)	0.0045 (9)
C1	0.0555 (14)	0.0541 (14)	0.0476 (13)	−0.0105 (11)	0.0112 (11)	−0.0020 (11)
C1'	0.0517 (13)	0.0397 (12)	0.0506 (13)	0.0065 (10)	0.0095 (10)	−0.0031 (10)
C2	0.0425 (11)	0.0498 (13)	0.0456 (12)	−0.0012 (10)	0.0137 (10)	0.0001 (10)
C2'	0.0402 (11)	0.0408 (12)	0.0546 (13)	−0.0012 (9)	0.0120 (10)	−0.0048 (10)
C3	0.0367 (10)	0.0510 (13)	0.0449 (12)	−0.0048 (9)	0.0110 (9)	0.0004 (10)
C3'	0.0352 (10)	0.0391 (11)	0.0519 (12)	0.0022 (9)	0.0066 (9)	−0.0029 (9)
C4	0.0374 (11)	0.0491 (13)	0.0504 (12)	−0.0006 (9)	0.0113 (9)	0.0100 (10)
C4'	0.0379 (11)	0.0395 (11)	0.0515 (12)	0.0002 (9)	0.0109 (9)	−0.0070 (10)
C5	0.0384 (12)	0.0568 (15)	0.0643 (15)	0.0003 (10)	0.0077 (11)	0.0135 (12)
C5'	0.0364 (11)	0.0449 (12)	0.0614 (14)	0.0004 (9)	0.0087 (10)	−0.0089 (10)
C6	0.0425 (12)	0.0654 (16)	0.0598 (15)	−0.0136 (11)	−0.0003 (11)	0.0053 (13)
C6'	0.0397 (11)	0.0505 (14)	0.0610 (14)	0.0076 (10)	0.0011 (10)	−0.0072 (11)
C7	0.0382 (11)	0.0536 (13)	0.0471 (12)	0.0056 (10)	0.0116 (9)	0.0109 (10)
C7'	0.0351 (10)	0.0414 (12)	0.0504 (12)	−0.0035 (9)	0.0107 (9)	−0.0060 (9)
C8	0.0499 (13)	0.0579 (15)	0.0592 (15)	0.0042 (11)	0.0090 (11)	−0.0081 (12)
C8'	0.0456 (12)	0.0432 (12)	0.0568 (14)	0.0009 (10)	0.0096 (10)	0.0064 (10)
C9	0.0385 (11)	0.0514 (14)	0.0693 (17)	0.0001 (10)	0.0162 (11)	−0.0076 (12)
C9'	0.0349 (10)	0.0453 (12)	0.0553 (14)	0.0019 (9)	0.0096 (10)	0.0067 (10)
C10	0.088 (2)	0.0523 (17)	0.130 (3)	−0.0070 (16)	0.050 (2)	0.0034 (18)
C10'	0.082 (2)	0.0484 (15)	0.103 (2)	−0.0009 (14)	0.0353 (18)	−0.0128 (15)
C11	0.092 (2)	0.074 (2)	0.101 (3)	−0.0303 (18)	0.005 (2)	−0.0312 (19)
C11'	0.091 (2)	0.0580 (17)	0.0742 (19)	0.0170 (16)	−0.0049 (17)	0.0139 (14)

### Geometric parameters (Å, º)

**Table:**

O1—C1	1.341 (3)	C3—H3	0.9300
O1—C11	1.424 (3)	C3'—C4'	1.385 (3)
O1'—C1'	1.340 (3)	C3'—H3'	0.9300
O1'—C11'	1.424 (3)	C4—C5	1.396 (3)
O2—N1	1.197 (3)	C4—C7	1.489 (3)
O2'—N1'	1.197 (3)	C4'—C5'	1.389 (3)
O3—N1	1.200 (3)	C4'—C7'	1.494 (3)
O3'—N1'	1.213 (3)	C5—C6	1.371 (4)
O4—C7	1.233 (3)	C5—H5	0.9300
O4'—C7'	1.231 (2)	C5'—C6'	1.374 (3)
O5—C9	1.192 (3)	C5'—H5'	0.9300
O5'—C9'	1.195 (3)	C6—H6	0.9300
O6—C9	1.332 (3)	C6'—H6'	0.9300
O6—C10	1.440 (4)	C8—C9	1.496 (4)
O6'—C9'	1.328 (3)	C8—H8A	0.9700
O6'—C10'	1.438 (3)	C8—H8B	0.9700
N1—C2	1.461 (3)	C8'—C9'	1.499 (3)
N1'—C2'	1.463 (3)	C8'—H8'A	0.9700
N2—C7	1.343 (3)	C8'—H8'B	0.9700
N2—C8	1.439 (3)	C10—H10D	0.9600
N2—H2	0.828 (16)	C10—H10E	0.9600
N2'—C7'	1.341 (3)	C10—H10F	0.9600
N2'—C8'	1.440 (3)	C10'—H10A	0.9600
N2'—H2'	0.833 (16)	C10'—H10B	0.9600
C1—C6	1.395 (4)	C10'—H10C	0.9600
C1—C2	1.407 (3)	C11—H11D	0.9600
C1'—C6'	1.394 (3)	C11—H11E	0.9600
C1'—C2'	1.407 (3)	C11—H11F	0.9600
C2—C3	1.378 (3)	C11'—H11A	0.9600
C2'—C3'	1.375 (3)	C11'—H11B	0.9600
C3—C4	1.389 (3)	C11'—H11C	0.9600
			
C1—O1—C11	118.7 (2)	C5'—C6'—H6'	119.7
C1'—O1'—C11'	118.7 (2)	C1'—C6'—H6'	119.7
C9—O6—C10	117.4 (2)	O4—C7—N2	121.9 (2)
C9'—O6'—C10'	117.7 (2)	O4—C7—C4	121.0 (2)
O2—N1—O3	121.1 (2)	N2—C7—C4	117.11 (19)
O2—N1—C2	120.6 (2)	O4'—C7'—N2'	122.3 (2)
O3—N1—C2	118.0 (2)	O4'—C7'—C4'	121.19 (19)
O2'—N1'—O3'	121.9 (2)	N2'—C7'—C4'	116.47 (18)
O2'—N1'—C2'	120.5 (2)	N2—C8—C9	113.5 (2)
O3'—N1'—C2'	117.2 (2)	N2—C8—H8A	108.9
C7—N2—C8	122.0 (2)	C9—C8—H8A	108.9
C7—N2—H2	122.2 (18)	N2—C8—H8B	108.9
C8—N2—H2	115.6 (18)	C9—C8—H8B	108.9
C7'—N2'—C8'	122.38 (19)	H8A—C8—H8B	107.7
C7'—N2'—H2'	122.0 (17)	N2'—C8'—C9'	113.4 (2)
C8'—N2'—H2'	115.0 (17)	N2'—C8'—H8'A	108.9
O1—C1—C6	124.5 (2)	C9'—C8'—H8'A	108.9
O1—C1—C2	118.1 (2)	N2'—C8'—H8'B	108.9
C6—C1—C2	117.4 (2)	C9'—C8'—H8'B	108.9
O1'—C1'—C6'	124.7 (2)	H8'A—C8'—H8'B	107.7
O1'—C1'—C2'	118.3 (2)	O5—C9—O6	125.0 (3)
C6'—C1'—C2'	116.9 (2)	O5—C9—C8	125.1 (2)
C3—C2—C1	121.5 (2)	O6—C9—C8	110.0 (2)
C3—C2—N1	117.0 (2)	O5'—C9'—O6'	125.3 (2)
C1—C2—N1	121.5 (2)	O5'—C9'—C8'	125.5 (2)
C3'—C2'—C1'	122.2 (2)	O6'—C9'—C8'	109.2 (2)
C3'—C2'—N1'	116.83 (19)	O6—C10—H10D	109.5
C1'—C2'—N1'	121.0 (2)	O6—C10—H10E	109.5
C2—C3—C4	120.8 (2)	H10D—C10—H10E	109.5
C2—C3—H3	119.6	O6—C10—H10F	109.5
C4—C3—H3	119.6	H10D—C10—H10F	109.5
C2'—C3'—C4'	120.1 (2)	H10E—C10—H10F	109.5
C2'—C3'—H3'	119.9	O6'—C10'—H10A	109.5
C4'—C3'—H3'	119.9	O6'—C10'—H10B	109.5
C3—C4—C5	117.5 (2)	H10A—C10'—H10B	109.5
C3—C4—C7	123.7 (2)	O6'—C10'—H10C	109.5
C5—C4—C7	118.8 (2)	H10A—C10'—H10C	109.5
C3'—C4'—C5'	118.1 (2)	H10B—C10'—H10C	109.5
C3'—C4'—C7'	122.56 (19)	O1—C11—H11D	109.5
C5'—C4'—C7'	119.31 (19)	O1—C11—H11E	109.5
C6—C5—C4	122.2 (2)	H11D—C11—H11E	109.5
C6—C5—H5	118.9	O1—C11—H11F	109.5
C4—C5—H5	118.9	H11D—C11—H11F	109.5
C6'—C5'—C4'	122.0 (2)	H11E—C11—H11F	109.5
C6'—C5'—H5'	119.0	O1'—C11'—H11A	109.5
C4'—C5'—H5'	119.0	O1'—C11'—H11B	109.5
C5—C6—C1	120.5 (2)	H11A—C11'—H11B	109.5
C5—C6—H6	119.7	O1'—C11'—H11C	109.5
C1—C6—H6	119.7	H11A—C11'—H11C	109.5
C5'—C6'—C1'	120.5 (2)	H11B—C11'—H11C	109.5
			
C11—O1—C1—C6	−3.9 (4)	C3'—C4'—C5'—C6'	1.6 (3)
C11—O1—C1—C2	177.3 (3)	C7'—C4'—C5'—C6'	−178.4 (2)
C11'—O1'—C1'—C6'	−1.0 (4)	C4—C5—C6—C1	2.2 (4)
C11'—O1'—C1'—C2'	177.4 (2)	O1—C1—C6—C5	179.4 (2)
O1—C1—C2—C3	178.6 (2)	C2—C1—C6—C5	−1.9 (4)
C6—C1—C2—C3	−0.2 (3)	C4'—C5'—C6'—C1'	−2.2 (4)
O1—C1—C2—N1	−2.8 (3)	O1'—C1'—C6'—C5'	179.2 (2)
C6—C1—C2—N1	178.3 (2)	C2'—C1'—C6'—C5'	0.9 (4)
O2—N1—C2—C3	159.7 (3)	C8—N2—C7—O4	−1.6 (4)
O3—N1—C2—C3	−13.9 (4)	C8—N2—C7—C4	178.6 (2)
O2—N1—C2—C1	−18.9 (4)	C3—C4—C7—O4	−160.1 (2)
O3—N1—C2—C1	167.5 (3)	C5—C4—C7—O4	18.0 (3)
O1'—C1'—C2'—C3'	−177.4 (2)	C3—C4—C7—N2	19.8 (3)
C6'—C1'—C2'—C3'	1.0 (3)	C5—C4—C7—N2	−162.1 (2)
O1'—C1'—C2'—N1'	2.4 (3)	C8'—N2'—C7'—O4'	3.0 (4)
C6'—C1'—C2'—N1'	−179.1 (2)	C8'—N2'—C7'—C4'	−176.1 (2)
O2'—N1'—C2'—C3'	−148.6 (3)	C3'—C4'—C7'—O4'	157.6 (2)
O3'—N1'—C2'—C3'	24.8 (4)	C5'—C4'—C7'—O4'	−22.4 (3)
O2'—N1'—C2'—C1'	31.6 (4)	C3'—C4'—C7'—N2'	−23.3 (3)
O3'—N1'—C2'—C1'	−155.0 (3)	C5'—C4'—C7'—N2'	156.7 (2)
C1—C2—C3—C4	2.1 (3)	C7—N2—C8—C9	−88.1 (3)
N1—C2—C3—C4	−176.5 (2)	C7'—N2'—C8'—C9'	87.3 (3)
C1'—C2'—C3'—C4'	−1.6 (4)	C10—O6—C9—O5	4.3 (4)
N1'—C2'—C3'—C4'	178.5 (2)	C10—O6—C9—C8	−175.3 (2)
C2—C3—C4—C5	−1.8 (3)	N2—C8—C9—O5	13.9 (4)
C2—C3—C4—C7	176.4 (2)	N2—C8—C9—O6	−166.5 (2)
C2'—C3'—C4'—C5'	0.3 (3)	C10'—O6'—C9'—O5'	−7.6 (4)
C2'—C3'—C4'—C7'	−179.7 (2)	C10'—O6'—C9'—C8'	172.9 (2)
C3—C4—C5—C6	−0.3 (4)	N2'—C8'—C9'—O5'	−10.9 (3)
C7—C4—C5—C6	−178.6 (2)	N2'—C8'—C9'—O6'	168.53 (18)

### Hydrogen-bond geometry (Å, º)

**Table:**

*D*—H···*A*	*D*—H	H···*A*	*D*···*A*	*D*—H···*A*
N2—H2···O4′^i^	0.83 (2)	2.26 (2)	3.069 (2)	167 (2)
N2′—H2′···O4	0.83 (2)	2.23 (2)	3.016 (2)	158 (2)

Symmetry code: (i) *x*+1, *y*, *z*.
